# Appendicectomy for Uncomplicated Simple Appendicitis: Is It Always Required?

**DOI:** 10.1155/2021/8848162

**Published:** 2021-03-15

**Authors:** Ibrahim Falih Noori Alsubsiee, Ahmed Falih Noori Alsubsiee

**Affiliations:** ^1^Department of Surgery, College of Medicine, Basra University, Basra, Iraq; ^2^Basra Health Directorate, Basra, Iraq

## Abstract

**Background:**

Although appendicectomy is still the classical and standard treatment for acute appendicitis, initial conservative antibiotic only treatment for simple uncomplicated cases has been proposed and tried as a feasible and effective approach. The objective of this study was to evaluate the efficacy and outcomes of antibiotics treatment for acute simple uncomplicated appendicitis.

**Methods:**

This is a prospective controlled nonrandomized study in which a total of 156 patients whose ages range from 16 to 54 years presenting with clinical diagnosis of acute uncomplicated appendicitis were assigned for conservative antibiotics treatment, which consists of ceftriaxone I gram twice daily and metronidazole infusions, 500 mg in 100 ml, 3 times daily for 48 to 72 hours to be converted on oral antibiotics after clinical improvement for 5 to 7 days. Patients who failed to initial conservative treatment and those who had recurring symptoms of appendicitis were presented for appendectomy.

**Results:**

Antibiotic treatment was successful and feasible in 138 (88.5%) patients. Progression of the signs and symptoms despite full medical treatment was observed in 11 (7%) patients during the same admission. Further 7 (4.5%) patients showed recurrence of the symptoms during follow-up period of 6–12 months after successful initial conservative treatment and also proceeded for appendicectomy.

**Conclusion:**

Nonoperative antibiotic treatment of acute simple appendicitis is safe, feasible, and effective for properly selected cases, thus avoiding unnecessary surgery with its possible complications.

## 1. Introduction

Acute appendicitis is the most frequent emergency in the general surgical practice worldwide. The life-time incidence of acute appendicitis is estimated to be one in ten people. Surgery in form of appendicectomy has remained the standard classical urgent or emergent procedure of choice for decades to avoid the progressive inflammation that leads ultimately to perforation [1]. It has been found recently that such progressive nature of acute appendicitis and perforation is quite uncommon, especially in young and adult patients, and the majority of the cases are simple and uncomplicated [1, 2]. Recently, there have been increasing and ongoing debates about the role of the conservative nonoperative treatment of uncomplicated acute appendicitis using specific antibiotics and supportive measures. Conservative nonsurgical treatment of certain intra-abdominal inflammation such as salpingitis, diverticulitis, and inflammatory bowel diseases is a well-established and valid treatment modality. Although appendicectomy is simple and safe procedure, it can result in several complications such as wound infection, pelvic abscess, bowel obstruction due to adhesion, pneumonia, and enterocutaneous fistula [3]. There has been growing evidence and trend toward primary antibiotic treatment that has gained more acceptance in the last few years for selected patients with uncomplicated acute appendicitis. Several studies and researches have been published in attempt to evaluate the effectiveness, safety, and the outcomes of the conservative management. The results, however, are still controversial and general consensus is still lacking. The main purpose of this work was to assess the effectiveness and feasibility of antibiotic conservative approach as the sole treatment modality of simple uncomplicated appendicitis in terms of short-term and long-term outcomes, complication, length of the hospital stay, sick leave, and overall effectiveness.

## 2. Patients and Methods

This is a prospective controlled study conducted in one major hospital for the period between August 2016 and February 2020 in which a total of 156 patients whose ages range from 16 to 54 years presenting with clinical diagnosis of acute uncomplicated appendicitis were assigned for conservative antibiotics treatment. The diagnosis of acute appendicitis was made by detailed history of mild-to-moderate right lower abdominal pain associated with nausea and anorexia and carful clinical exam of localized and rebound tenderness in the right iliac fossa. The definite diagnosis of acute appendicitis was confirmed by laboratory blood investigations, mainly CBC and C-reactive protein and imaging (ultrasound and CT scan), which were done for all patients enrolled in this study. Alvarado scores of all patients were obtained for assured diagnosis (Table 1). Pregnancy test was done for all female patients.

The treatment modality has been fully explained to all patients and written informed consents were obtained. The study was conducted after approval of ethical committee of College of Medicine, University of Basra, Iraq. The inclusion criteria of the participants in this study were all those patients aged above 16 years with clinical diagnosis of acute appendicitis made by senior or senior house officer surgeons confirmed by validated Alvarado score [4] ≤6, elevated blood inflammatory markers (WBCs, neutrophilia, and elevated C-reactive protein), and imaging mainly by high-resolution ultrasound and CT scan.

Patients with severe acute complicated appendicitis such as perforation, abscess, and localized, or diffused peritonitis, those with comorbidities such as diabetes, congenital hemolytic anaemia, and hypertension, and those with low immunity and history of allergy to antibiotics as well as those who refused conservative treatment and preferred surgery were excluded. Informed written consent was obtained from all patients who were enrolled in this study. Female patients with positive pregnancy test were also excluded.

All patients included in this study were admitted to the surgical ward and asked to be nil by mouth and received intravenous fluid. Patients then received parenteral antibiotics (ceftriaxone I gram twice daily and metronidazole infusions, 500 mg in 100 ml, 3 times daily for 48 to 72 hours. The patients were regularly monitored by 12 hourly charts, which included vital signs, localized abdominal signs, and symptoms changes. Intravenous ciprofloxacin in a dose of 400 mg twice daily was used for patients allergic to cephalosporin (6 patients). Patients whose conditions got improved both clinically and by investigations were discharged home on oral antibiotics (cefixime 400 mg twice daily or ciprofloxacin 500 mg three times daily with metronidazole 500 mg three times a day for 7 to 10 days) to be seen after that for further checking and evaluation. During hospital stay, patients whose symptoms and signs showed no improvement or even worsened were proceeded for appendicectomy (11 patients).

Patients who showed successful conservative treatment were informed to come back if their initial symptoms recurred. All patients treated conservatively were followed up for 6 to 12 months. The main objectives of this study are to determine the feasibility and outcomes of antibiotics conservative treatment for uncomplicated acute appendicitis. The primary end-point of this work was to identify the number of patients with successful complications-free conservative treatment as being discharged from hospital after complete resolutions of their signs and symptoms with no need for appendicectomy and no recurrence of the same symptoms during the follow-up period. The second end-point was to assess the length of hospital stay, evaluations of the pain using the visual analogue scale, return to normal activity, sick-leave period, and return to normal life as well as the cost of conservative treatment compared with surgical interventions. Statistical analysis of the data was done using IBM SPSS version 22. Chi-square test was used to determine the significance association between the variables. *P* value < 0.05 was deemed significant.

## 3. Results

A total of 156 patients with acute uncomplicated appendicitis were assigned to be managed conservatively. Their ages range between 16 and 54 years, with mean of 36.8 years. They consist of 80 males (51.3%) and 76 females (48.7%), so the sex ratio was comparable. The highest incidence of acute appendicitis was among age groups ≥16–25 and 26–25 : 46 patients (29.5%) and 55 patients (35.3%), respectively (Table 2).

Regarding the presentation of the patients to the hospital with signs and symptoms of acute appendicitis, 102 patients (65.4%) presented in less than 24 hours, 34 patients (21.8%) presented within 24–48 hours, and 20 patients (12.8%) presented within 48–72-hour duration (Table 3).

The diagnosis of acute uncomplicated appendicitis depends on history and clinical exam, laboratory investigations, mainly the inflammatory markers triad (leukocytosis, neutrophilia, and C-reactive protein), and imaging, mainly ultrasound, which was done for all patients and CT scan which was done only for query cases (46 patients).

Among 156 patients who were selected to be managed conservatively with antibiotics, this treatment was successful and feasible in 138 (88.5%) patients (75 males and 63 females). Progression of the signs and symptoms despite full medical treatment was observed in 11 (7%) patients during the same admission and therefore they were submitted to appendicectomy. Further 7 (4.5%) patients showed recurrence of the symptoms during follow-up period of 6–12 months after successful initial conservative treatment and also proceeded for appendicectomy (Table 4).

## 4. Discussion

Acute appendicitis is the most common cause of acute abdomen with estimated incidence of about one in ten people during their lifetime. The majority of the cases presented as simple and uncomplicated [2, 4]. The process of acute appendicitis was considered as progressive which might lead to perforation with localized or diffuse peritonitis if not treated in proper time frame [6, 7]; such scenario, however, was found to be uncommon. Although appendicectomy remained the traditional gold-standard treatment for decades, such operation, however, has significant adverse short-term and long-term complications such as wound infection, enterocutaneous fistula, and adhesive small bowel obstruction requiring surgery and tubal infertility in females. Furthermore, about 15–30% of surgical explorations result in negative appendicectomies [7].

Recently, with advanced preoperative diagnostic facilities, in particular improvement in the diagnostic imaging using high-resolution ultrasound and abdominal CT scan, the diagnosis of simple and uncomplicated acute appendicitis can be confidently established. Recently, there has been an increasing trend for the treatment of simple appendicitis conservatively using antibiotics depending upon several pathophysiological and radiological lines of evidence which was no longer considered acute simple appendicitis as invariably progressive disease. Therefore, several authors have recently suggested conservative antibiotics as primary treatment of acute simple appendicitis [6, 8–12].

We found in our study that the success rate of antibiotics treatment among 156 patients with simple acute appendicitis was 88.5% (138 patients, 75 males and 63 females). 11 patients (7%) showed progression of the signs and symptoms despite full medical treatment during the same admission and therefore they were submitted to appendicectomy. Further 7 (4.5%) patients showed recurrence of the symptoms during follow-up period of 6–12 months after successful initial conservative treatment and also proceeded for appendicectomy. So, a total of 18 patients failed to respond, resulting in a failure rate of 11.5%. Thus, nonoperative antibiotic management could be feasible and successful alternative in selected patients with uncomplicated appendicitis who accept some probable risk of recurrence.

The main advantages of antibiotics treatment are that it is an effective and feasible alternative to treat acute appendicitis when surgery is contraindicated or not accessible or even when patients refuse surgery and the complications rate is less than that of appendicectomy; the hospital stay, the sick leave, and cost-effectiveness of nonoperative treatment are significantly shorter compared with appendectomy. On the other hand, the main drawbacks of antibiotic treatment are the risk of recurrent disease which could be as high as 35%, lack of definite histopathology, and the probable increase in antibiotic resistance and *Clostridium difficile* infections [13].

The conservative approach is usually entailed in hospital initial course of intravenous antibiotics that consist of cephalosporin and metronidazole for 48 to 72 hours followed by 5 to 7 days' course of oral antibiotics that include metronidazole and oral cephalosporin or ciprofloxacin. The first parenteral antibiotics should be given in hospital to assess the response to the treatment and to perform appendicectomy if the condition is worsening.

It has been found that several factors that present at admission are considered to be independent predictors of successful antibiotics treatment of acute simple appendicitis including low grade fever, low concentration of C-reactive protein, lower modified Alvarado score [14] (≤6), and smaller diameter of the appendix with no appendicolith by imaging. Further, patients with a longer duration of symptoms were more likely to have a successful conservative treatment [15, 16]. In fact, the use of Adult Appendicitis Score (AAS), modified Alvarado score, and Appendicitis Inflammatory Response score (AIR score) as clinical predictors of acute appendicitis is a cost-effective method to reduce the negative appendectomy rate and can stratify patients into high-risk group with specificity of up to 94%. There are currently two principal management strategies for patients with suspected appendicitis: the score-based risk stratification followed by a diversified management depending on the estimated risk of appendicitis and the imaging-based strategy advocating routine diagnostic imaging in all patients except those who were deemed to have low probability of appendicitis. In patients below 40 years of age who have been scored for high probability for acute appendicitis (AIR score 9–12, Alvarado score 9–10, and AAS ≥16), CT scan adds little value in the diagnosis. A high-probability score of acute appendicitis may be used to select patients below 40 yearsin which imaging is not needed [15].

Several studies have been published in the last few years regarding only the conservative antibiotics of acute simple appendicitis. Rollins et al. [17] in their five randomized controlled trials with total of 1430 patients in which 727 proceeded for antibiotics conservative treatment and 703 underwent appendicectomy showed that there was a 39% risk reduction in overall complication rate in the antibiotic group compared with those undergoing appendicectomy. There was no significant difference in duration of hospital stay. In conservative group patients, 21% patients (123 out of 587) initially treated conservatively with antibiotics were readmitted with symptoms of recurrent appendicitis and appendicectomy was done for all of them. The rate of complicated appendicitis was not increased in patients who underwent appendectomy after failed antibiotic management (10.8%) versus those who had primary appendicectomy (17.9%) and, even for complicated recurrent appendicitis after failed conservative treatment, a low-cost, cost-effective, and safe laparoscopic appendectomy is still feasible versus an open appendectomy, either with a traditional laparoscopy or even a single-incision laparoscopic surgery (SILS) [18]. Lui et al. [19] in a meta-analysis and systemic review of the use of antibiotics alone for treatment of acute appendicitis included a total of 1201 patients and recorded a success rate of 93.1% and a rate of recurrent appendicitis of 14.2%, while Wilms et al. [20] in their systemic review found that appendicectomy remains the treatment of choice for acute appendicitis due to high success rate of 97.4% compared with 73.4% for patients treated with antibiotics alone.

Harnoss et al. [21] in their four trials and four cohort studies that included 2551 patients found that the effectiveness of conservative antibiotic treatment of acute appendicitis was 72.6%, significantly lower than the 99.4% in the appendicectomy group with 26.5% of patients treated conservatively needing appendicectomy within 1 year. The overall postoperative complications were comparable. The hospital stay was significantly higher in the antibiotic treatment group in randomized trials. Similar results were obtained by Mumtaz et al. [22] in their single-hospital-based prospective study of 90 patients with simple uncomplicated appendicitis. They found that conservative treatment was successful in 75.6% patients. They concluded that the majority of simple, acute appendicitis cases can be treated effectively by antibiotics treatment.

Saverio et al. [23] in their NOTA study (Nonoperative Treatment for Acute Appendicitis) concluded that antibiotic treatment for simple noncomplicated acute appendicitis is safe and effective and could avoid unnecessary surgery, decreasing operation rate, surgical risk, and overall cost. They found that the recurrences of the symptoms after 2 years' follow-up period were less than 14% and may be safely and effectively treated with antibiotics also.

It is worth noting that colonic screening with colonoscopy and/or enhanced CT scan for those patients more than 40 years old with appendicitis is recommended, since the incidence of appendicular neoplasms is relatively high [24].

The long-term outcome of conservative treatment of appendicitis represented by recurrence is a major concern. Our results showed that the recurrence rate after 6 to 12 months' follow-up period was 4.5%. All the recurrent cases were simple and no perforation or abscess was detected and they were managed successfully by appendicectomy with no complications or mortality. Lundholm et al. [25] found that the risk of long-term relapse of antibiotics treatment of acute appendicitis was around 15% following successful initial conservative treatment, which may imply an overall benefit of 60–70% by conservative treatment during the long-term follow-up period of 10 years. McCutcheon et al. [26] and Tanaka et al. [27] recorded recurrence rates of 4.4% and 28.6%, respectively. Further, a similar study by Salminen et al. [28] showed that the likelihood of the late recurrence among patients who were initially treated successfully with antibiotics within 5 years was 39.1%. These findings support the feasibility of conservative antibiotic treatment alone as alternative to appendicectomy.

Ielpo et al. [29] in their online survey designed by the Association of Italian Surgeons in Europe online survey to assess the current attitude of surgeons globally regarding the management of patients with acute appendicitis during the pandemic that found approximately 22 percent of the respondents declared that they would change their attitude from surgery to conservative treatment with antibiotics, or vice versa, if they had the chance to test all patients before surgery; 17.5 percent stated that they already tested all patients, whereas 26.9 percent stated that they would have changed their attitude only if quick tests or PCR was available.

Before the COVID‐19 pandemic, 6.6 percent of the respondents adopted nonoperative treatment with antibiotics for patients with uncomplicated acute appendicitis, compared with 23.7 percent during the pandemic (*P* < 0.001). Regarding complicated acute appendicitis, nonoperative treatment was used by 2.4 and 5.3 percent before and during the pandemic and percutaneous drainage by 21.1 *versus* 32.9 percent, respectively.

Patients' selection for conservative treatments may not be an easy task even when using both clinical scores and CT scan, with the latter being used more often recently. David et al. [30] reported that medical imaging, whether ultrasound or CT scan, had a 70% prediction rate for acute appendicitis, 20% false-negative rate, and 10% false-positive rate. They further recorded that the overall prediction rate for appendicitis by clinical assessment supplemented by laboratory tests and imaging was 93.2%. Lastunen et al. [31] reported that a substantial proportion of patients with uncomplicated acute appendicitis on CT have complicated appendicitis at surgery. However, in patients with no risk factors, surgery can be postponed safely for up to 7 hours. It must be emphasized that several patients may present CT findings suggesting appendicitis but do not have an actual acute appendicitis. Conversely, patients with a clinically clear acute appendicitis may not have a visible appendix on CT scan. Thus, the clinical evaluation is still paramount to the management of patients with suspected acute appendicitis before considering medical imaging.

## 5. Conclusion

Conservative antibiotic treatment of acute simple and uncomplicated appendicitis is safe and has high efficacy as most meta-analyses showed. With proper and strict selection of the patients, antibiotic treatment alone can be safely applied to the majority of the patients who presented with first attack of uncomplicated appendicitis. Although appendicectomy is still more effective than conservative treatment, the rate of complications is significantly lower in conservative treatment. The length of hospital stay was higher in conservative approach but the return to normal daily activities and sick leave and overall cost were less in antibiotics treatment. The main concerns of conservative treatment are the risk of the treatment failure and recurrence of symptoms, the probable increase in antibiotic resistance, and lack of definite histopathology. Therefore, conservative antibiotics alone could be considered as an efficient primary treatment for acute simple appendicitis, reserving an appendicectomy only for those patients who failed to respond to antibiotic treatment and for recurrent cases.

## Figures and Tables

**Table 1:** Alvarado score for diagnosis of acute appendicitis^*∗*^.

Feature	Score
Migratory pain	1
Anorexia	1
Nausea	1
Tenderness in right lower quadrant	2
Rebound pain	1
Elevated temperature	1
Leukocytosis	2
Shift of white blood cells count to the left	1

Total	10

^*∗*^Alvarado score: 0–4: unlikely appendicitis; 5-6: equivocal for appendicitis; 7-8: probably appendicitis; 9-10: most likely appendicitis (Table 1 is from Neupane et al. [5] under the Creative Commons Attribution License/Public Domain).

**Table 2:** Age distribution of the patients included in the study.

Age	Number of patients		Mean age
	Males	Females	
**16–20**	12	17	**29**
21–30	24	21	45
31–40	32	26	58
41–50	6	8	14
51–60	6	4	10

Total	80 (51.3%)	76 (48.7%)	156 (mean: 36.8)

**Table 3:** Distribution of the patients according to duration of the symptoms.

Duration of the symptoms (hours)	Number of patients		Total (%)
	Males	Females	
≤24	44	40	88 (56.4)
24–48	26	23	45 (28.9)
48–72	10	13	23 (14.7)

Total	80	76	156

**Table 4:** Outcomes of conservative antibiotic treatment of simple and uncomplicated appendicitis.

Overall outcomes	Outcomes of conservative antibiotics treatment		
		Success	Initial failure	Recurrence
		138 (88.5%)	11 (7%)	7 (4.5%)
According to sex	Male	75 (93.75%)	4 (5%)	5 (6.25%)
	Female	63 (82.9%)	7 (9.2%)	2 (2.6%)

According to age (years)	≤20	26 (89.7%)	2 (6.9%)	1 (3.4%)
	21–30	40 (88.9%)	3(6.7%)	2 (4.4%)
	31–40	51 (87.9%)	4 (6.9%)	3 (5.2%)
	41–50	12 (85.8%)	1 (7.1%)	1(7.1%)
	51–60	9 (90%)	1 (10%)	0 (0%)

According to duration of symptoms (hours)	≤24	85 (96.6%)	2 (2.3%)	1 (1.1%)
	24–48	38 (84.4%)	4 (8.9%)	3 (6.7%)
	48–72	15 (65.2%)	5 (21.7%)	3 (13.1%)

## Data Availability

The data are available upon request from the corresponding author.
